# The Tiotropium Safety and Performance in Respimat® (TIOSPIR®) Trial: Spirometry Outcomes

**DOI:** 10.1186/s12931-015-0269-4

**Published:** 2015-09-15

**Authors:** Antonio Anzueto, Robert Wise, Peter Calverley, Daniel Dusser, Wenbo Tang, Norbert Metzdorf, Ronald Dahl

**Affiliations:** Pulmonary/Critical Care, University of Texas Health Science Center, and South Texas Veterans Health Care System, 111E, 7400 Merton Minter Blvd, San Antonio, TX 78229 USA; Johns Hopkins University School of Medicine, Baltimore, MD USA; Institute of Ageing and Chronic Disease, University of Liverpool, Liverpool, UK; Service de Pneumologie Hôpital Cochin, Paris, France; Boehringer Ingelheim Pharmaceuticals Inc, Ridgefield, CT USA; Boehringer Ingelheim Pharma GmbH & Co KG, Ingelheim, Germany; Odense University Hospital, Odense, Denmark

## Abstract

**Background:**

Tiotropium Safety and Performance in Respimat® (TIOSPIR®) compared the safety and efficacy of tiotropium Respimat® and tiotropium HandiHaler® in patients with chronic obstructive pulmonary disease (COPD). A prespecified spirometry substudy compared the lung function efficacy between treatment groups.

**Methods:**

TIOSPIR® was a large-scale, long-term (2.3-year), event-driven, randomized, double-blind, parallel-group trial of 17,135 patients with COPD. In the spirometry substudy, trough forced expiratory volume in 1 second (FEV_1_) and forced vital capacity (FVC) were measured at baseline and every 24 weeks for the duration of the trial.

**Results:**

The substudy included 1370 patients who received once-daily tiotropium Respimat® 5 μg (n = 461), 2.5 μg (n = 464), or tiotropium HandiHaler® 18 μg (n = 445). Adjusted mean trough FEV_1_ (average 24–120 weeks) was 1.285, 1.258, and 1.295 L in the Respimat® 5 μg, 2.5 μg, and HandiHaler® 18 μg groups (difference versus HandiHaler® [95 % CI]: −10 [−38, 18] mL for Respimat® 5 μg and, −37 [−65, −9] mL for Respimat® 2.5 μg); achieving noninferiority to tiotropium HandiHaler® 18 μg for tiotropium Respimat® 5 but not for 2.5 μg (prespecified analysis). Adjusted mean trough FVC was 2.590, 2.544, and 2.593 L in the Respimat® 5 μg, 2.5 μg, and HandiHaler® 18 μg groups. The rates of FEV_1_ decline over 24 to 120 weeks were similar for the three treatment arms (26, 40, and 34 mL/year for the tiotropium Respimat® 5-μg, 2.5-μg, and HandiHaler® 18-μg groups). The rate of FEV_1_ decline in GOLD I + II patients was greater than in GOLD III + IV patients (46 vs. 23 mL/year); as well as in current versus ex-smokers, in patients receiving combination therapies at baseline versus not, and in those experiencing an exacerbation during the study versus not.

**Conclusions:**

The TIOSPIR® spirometry substudy showed that tiotropium Respimat® 5 μg was noninferior to tiotropium HandiHaler® 18 μg for trough FEV_1_, but Respimat® 2.5 μg was not. Tiotropium Respimat® 5 μg provides similar bronchodilator efficacy to tiotropium HandiHaler® 18 μg with comparable rates of FEV_1_ decline. The rate of FEV_1_ decline varied based on disease severity, with a steeper rate of decline observed in patients with moderate airway obstruction.

**Trial registration:**

NCT01126437.

**Electronic supplementary material:**

The online version of this article (doi:10.1186/s12931-015-0269-4) contains supplementary material, which is available to authorized users.

## Background

Tiotropium is delivered via the Respimat® Soft Mist™ Inhaler (SMI; 5 μg once daily) or the HandiHaler® device (18 μg once daily). Both provide similar improvements in lung function, exacerbation outcomes, symptoms and health-related quality of life (HRQoL) compared with placebo in patients with chronic obstructive pulmonary disease (COPD) [1–4]. While a decrease in mortality was observed for tiotropium HandiHaler® versus placebo in the Understanding Potential Long-term Impacts on Function with Tiotropium (UPLIFT®) trial [4], a numerical increase in all-cause mortality was observed with tiotropium Respimat® versus placebo in a pooled analysis of Respimat® trials, in particular in patients with known cardiac rhythm disorders [5].

These observations led to the development of the Tiotropium Safety and Performance in Respimat® (TIOSPIR®) trial [6, 7], the first large-scale, long-term trial of tiotropium Respimat® and tiotropium HandiHaler® to compare directly the safety and efficacy of the two formulations in patients with COPD. The primary endpoints were risk of death and risk of first COPD exacerbation. TIOSPIR® showed that tiotropium Respimat® 2.5 or 5 μg once daily have a similar safety and exacerbation efficacy profile to tiotropium HandiHaler® 18 μg once daily in patients with COPD [7].

Although there is a wealth of spirometry data on tiotropium HandiHaler®, less information is available for tiotropium Respimat®, and data on the rates of lung function decline are lacking in particular [1, 2]. A prespecified spirometry substudy was performed on 1370 patients within the TIOSPIR® trial and showed that Respimat® 5 μg was noninferior to HandiHaler® for the trough forced expiratory volume in 1 second (FEV_1_), while Respimat® 2.5 μg was not [7].

In these predefined analyses, we assessed additional lung function outcomes from the spirometry substudy, including forced vital capacity (FVC) and annual rates of decline in FEV_1_ and FVC. We wished to determine whether these outcomes differed between the tiotropium HandiHaler® and Respimat® arms, and assessed if rates of decline differed by patient baseline characteristics or between subgroups of patients with Global Initiative for Chronic Obstructive Lung Disease (GOLD) Stages I + II (predominantly GOLD II, as described in the Results) or III + IV COPD.

## Methods

TIOSPIR® was a large-scale, long-term, event-driven, randomized, double-blind, parallel-group trial of 17,135 patients with COPD. Detailed study methodology has been reported previously [6, 7].

### Study population

Patients enrolled were aged ≥40 years, had a clinical diagnosis of COPD, ≥10 pack-years, smoking history, a post-bronchodilator FEV_1_/FVC ratio ≤0.70, and an FEV_1_ ≤ 70 % predicted. Patients with concomitant cardiac disease were included, except for patients with unstable or recent events (myocardial infarction within the last 6 months, hospitalization for class III or IV heart failure, or unstable or life-threatening arrhythmia requiring new treatment within the last 12 months). Patients with other clinically significant lung diseases or a COPD exacerbation within the last month, moderate or severe renal impairment, cancer requiring therapy within the last 5 years, or drug or alcohol abuse within the last year were excluded. All COPD medications except other inhaled anticholinergic agents were allowed.

### Study design

TIOSPIR® compared the safety and efficacy of once-daily Respimat® 5 μg (two inhalations of 2.5 μg) and 2.5 μg (two inhalations of 1.25 μg) with HandiHaler® 18 μg. Primary endpoints were risk of death (noninferiority, Respimat® 5 μg or 2.5 μg vs. HandiHaler®) and risk of first COPD exacerbation (superiority, Respimat® 5 μg vs. HandiHaler®). Patients were seen every 12 weeks with a final visit 30 days after the end of treatment. The study protocol has been described in detail elsewhere [6, 7].

The trial was performed in accordance with the provisions of the Declaration of Helsinki, and the study protocol and procedures were approved by relevant institutional review boards and ethics committees. All the patients provided written informed consent.

### Spirometry substudy

The spirometry substudy was a predefined analysis which covered the duration of the study. The objective was to demonstrate that there were no differences between the interventions by testing the noninferiority of Respimat® 2.5 and 5 μg versus HandiHaler® 18 μg.

In the spirometry substudy population, trough FEV_1_ and FVC were measured at baseline and every 24 weeks (±14 days) for the duration of the trial (Weeks 24, 48, 72, 96, 120, 144, 168). The pulmonary function tests (PFTs) were done in the clinic at a single time point (in triplicate) prior to the daily dosing of tiotropium, and approximately 24 hours after dosing on the previous day (between 7:00 and 10:00 am). Spirometers and their use, including daily calibration, met American Thoracic Society (ATS)/European Respiratory Society (ERS) criteria. The highest FEV_1_ and FVC values from an acceptable maneuver were recorded regardless of whether they came from different spirometric maneuvers or from the same maneuver (preferably with a maximum of five attempts, but up to eight attempts were allowed). Predicted normal FEV_1_ values were calculated for patients using the European Community for Coal and Steel (ECCS) equations [8].

A number of medication restrictions were set on pulmonary function days for the substudy. Short-acting beta-adrenergic bronchodilators could not be taken for at least 8 hours prior to PFTs, and long-acting beta-adrenergic bronchodilators or combination beta-adrenergic bronchodilator/inhaled steroid could not be taken for at least 24 hours prior to PFTs. The morning dose of inhaled steroids could not be taken prior to PFTs and the morning dose of the study medication could not be taken prior to test-day pre-dose PFT. Short-acting (twice-daily or more frequent administration) theophylline preparations required at least a 24-hour washout, while long-acting (once-daily) theophylline preparation required at least a 48-hour washout.

### Statistical analysis

Summary statistics are used for presentation of the demographic data.

Noninferiority testing of tiotropium Respimat® 5 and 2.5 μg compared with tiotropium HandiHaler® 18 μg for trough FEV_1_ (average 24–120 weeks) was predefined. Noninferiority testing on trough (i.e., morning pre-dose) FEV_1_ was based on a noninferiority delta of 50 mL, assuming a standard deviation of 225 mL. The sample size needed was estimated at 427 patients per group for 90 % power and one-sided α = 0.025. Rounding to 435 patients per group, 1305 patients was the target sample size for the substudy. There were no predefined tests for FVC_._

Trough FEV_1_ (24 to 120 weeks) was analyzed between treatment groups using a mixed model repeated measures (MMRM) model [9] with an autoregression-1 covariance structure and the Kenwood–Roger approximation to estimate denominator degrees of freedom. Analyses included the fixed terms for treatment, investigative site, visit, treatment-by-visit interaction, baseline FEV_1_, and baseline FEV_1_-by-visit interaction, and a random term for patient. Superiority tests were clarified to be two-sided with α = 0.05 rather than one-sided with α = 0.025.

Mean FEV_1_ and FVC are reported as absolute values, adjusted by investigative site, visit, treatment-by-visit interaction, baseline FEV_1_, and baseline FEV_1_-by-visit interaction, within the MMRM model.

In a post-hoc analysis, the annual rate of decline for FEV_1_, FVC, and FEV_1_/FVC by treatment was estimated using a MMRM model that included the fixed terms of treatment, visit, and treatment-by-visit interaction, and a random intercept and slope, using data from Week 24 until the end of the treatment period. Annual rate of decline by GOLD stage (treatment arms pooled) was estimated using a similar MMRM model that included the fixed terms of GOLD stage, visit, GOLD-by-visit interaction, and a random intercept and slope.

Forest plots (showing means and 95 % CIs) were created for the spirometry substudy population to show rates of decline in FEV_1_, FVC, and FEV_1_/FVC by patient baseline characteristic or on-study exacerbation, estimated using the same MMRM model for post-hoc analysis.

## Results

### Study patients

A total of 1370 patients from the total population participated in the spirometry substudy and received once-daily tiotropium Respimat® 5 μg (n = 461), 2.5 μg (n = 464), or tiotropium HandiHaler® 18 μg (n = 445). The majority of patients were classified as TIOSPIR® GOLD Stages II (n = 632; 46.1 %), III (n = 580; 42.3 %), or IV (n = 130; 9.5 %), with few patients in GOLD Stage I (n = 4; 0.3 %) (23 patients had an FEV_1_/FVC ≥70 %, and 1 patient was not classified) (Table 1).

**Table 1:** Baseline characteristics of the spirometry substudy

Characteristic	Tiotropium Respimat® 5 μg (n = 461)	Tiotropium Respimat® 2.5 μg (n = 464)	Tiotropium HandiHaler® 18 μg (n = 445)
Male sex, %	63.6	62.9	59.3
Age, years	65.3 ± 9.1	65.6 ± 9.1	65.8 ± 8.4
Current smoker, %	39.9	40.1	33.3
Smoking history, pack-year	49.7 ± 27.7	51.0 ± 28.6	50.1 ± 28.9
FEV_1_, L	1.256 ± 0.445	1.215 ± 0.479	1.203 ± 0.489
FEV_1_, % predicted^a^	49.31 ± 12.99	48.26 ± 13.47	48.28 ± 13.63
FVC, L	2.555 ± 0.743	2.517 ± 0.796	2.482 ± 0.858
FEV_1_/FVC ratio, %	49.5 ± 11.0	48.4 ± 11.5	48.6 ± 11.2
GOLD Stage, %			
FEV_1_/FVC ≥70 %	1.3	1.7	2.0
I	0.7	0.0	0.2
II	49.2	46.1	42.9
III	40.8	42.0	44.3
IV	8.0	10.1	10.3
Any respiratory medication, %	95.7	95.0	97.8
Anticholinergics	69.2	69.6	69.0
LABA^b^	66.6	66.2	67.9
ICS^b^	60.1	61.2	62.0

^a^Post-bronchodilator

^b^Used alone or in combination

*Abbreviations*: *COPD* chronic obstructive pulmonary disease, *FEV*_*1*_ forced expiratory volume in 1 second, *FVC* forced vital capacity, *GOLD* Global Initiative for Chronic Obstructive Lung Disease, *ICS* inhaled corticosteroid, *LABA* long-acting β2-agonist

Baseline demographics and use of respiratory medication were similar among the three treatment arms (Table 1). Baseline FEV_1_ and FVC were, however, slightly elevated in the Respimat® 5 μg arm, which also exhibited slightly less lung function impairment (with a higher proportion of patients in GOLD II, and a higher FEV_1_ % predicted compared with the other arms), though these differences are not clinically significant. Baseline characteristics within the substudy were mostly similar to those of the total TIOSPIR® population (Additional file 1: Table S1) [7]. There were, however, proportionally fewer male patients in the substudy, since the substudy was not conducted in Asian sites, which typically have proportionally more male patients. Substudy patients also had a longer smoking history and used slightly more respiratory medication at baseline than patients not included in the substudy.

Median exposure was 853, 852, and 854 days to tiotropium Respimat® 5 μg, 2.5 μg, and HandiHaler® 18 μg, respectively, with a total of 3135 patient-years of exposure to tiotropium within the substudy.

### Spirometry analysis

**Fig. 1:**
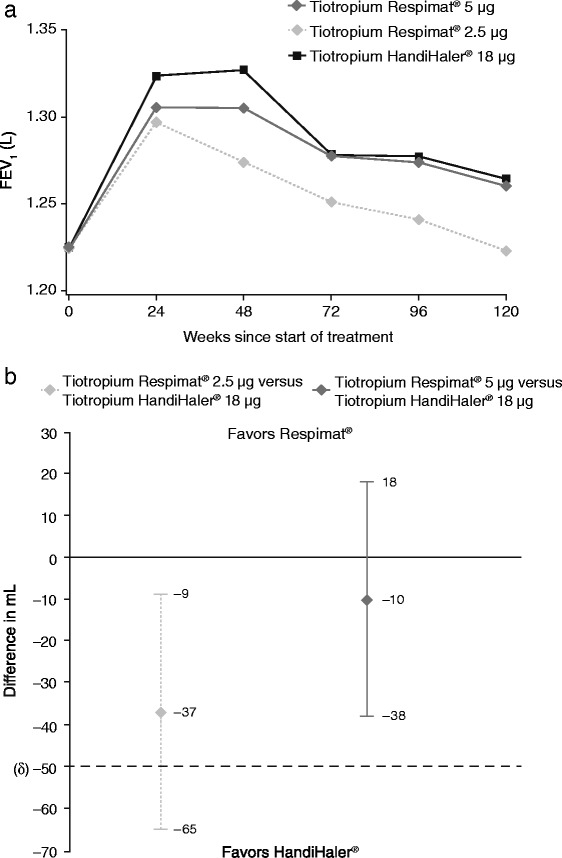
Trough forced expiratory volume in 1 second (FEV_1_) over time (**a**), and difference versus HandiHaler® 18 μg (**b**) [7]. Noninferiority was evaluated for treatment main effects using a noninferiority δ of 50 mL. *Abbreviations*: FEV_1_ = forced expiratory volume in 1 second

Over time, there was no difference in trough FEV_1_ between the tiotropium Respimat® 5 μg and HandiHaler® 18 μg treatment arms, with similar improvements from baseline that were greater than those obtained with tiotropium Respimat® 2.5 μg (Fig. 1a). As reported in the primary analysis [7], adjusted mean trough FEV_1_ (average 24–120 weeks) was similar for patients treated with tiotropium Respimat® 5 μg (1.285 L) and tiotropium HandiHaler® 18 μg (1.295 L; difference vs. HandiHaler®, −10 mL; 95 % confidence interval [CI], −38, 18 mL). Adjusted mean trough FEV_1_ (average 24 to 120 weeks) was 1.258 L for tiotropium Respimat® 2.5 μg (difference vs. HandiHaler®, −37 mL; 95 % CI −65, −9 mL). The adjusted mean trough FEV_1_ for tiotropium Respimat® 2.5 μg and 5 μg were 97.1 % and 99.2 % of the HandiHaler® 18 μg value. Noninferiority to tiotropium HandiHaler® 18 μg for trough FEV_1_ was therefore achieved for tiotropium Respimat® 5 μg but not for tiotropium Respimat® 2.5 μg (prespecified analysis) (Fig. 1b) [7].

**Fig. 2:**
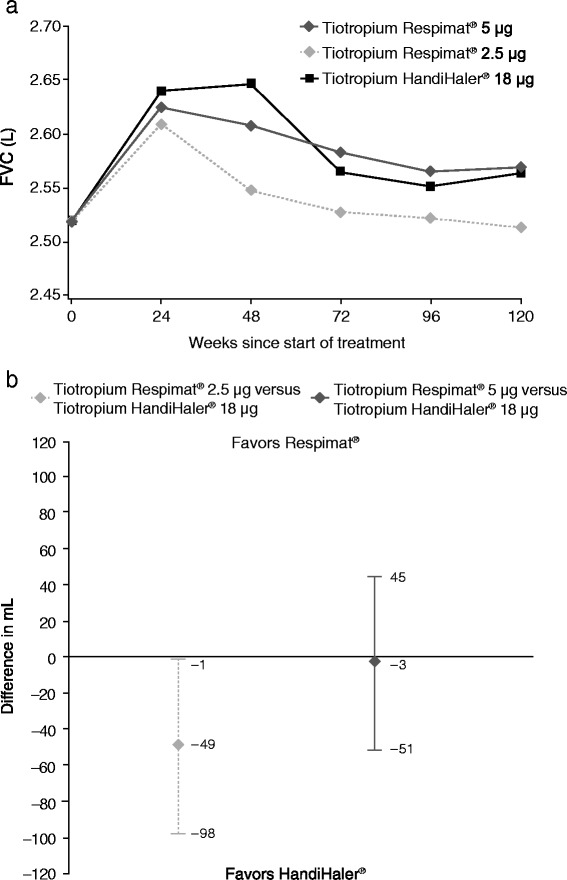
Trough forced vital capacity (FVC) over time (**a**), and difference versus HandiHaler® 18 μg (**b**). *Abbreviations*: FVC = forced vital capacity

In addition, tiotropium Respimat® 5 μg and HandiHaler® 18 μg also showed similar improvements in trough FVC over time that were greater than those obtained with tiotropium Respimat® 2.5 μg (Fig. 2a). Adjusted mean trough FVC (average 24–120 weeks) was similar for patients treated with tiotropium Respimat® 5 μg (2.590 L) and tiotropium HandiHaler® 18 μg (2.593 L) (difference vs. HandiHaler®, −3 mL; 95 % CI −51, 45 mL) (Fig. 2b). Adjusted mean trough FVC (average 24–120 weeks) was 2.544 L for tiotropium Respimat® 2.5 μg (difference vs. HandiHaler®, −49 mL; 95 % CI −98, −1 mL). The adjusted mean trough FVC for tiotropium Respimat® 2.5 μg and 5 μg were 98.1 % and 99.9 % of the HandiHaler® 18 μg value.

The rates of decline of FEV_1_ from 24 to 120 weeks were similar for the three treatment arms: 26, 40, and 34 mL/year for the tiotropium Respimat® 5 μg, 2.5 μg, and HandiHaler® 18 μg groups, respectively. These were not statistically different from each other (Table 2). The rates of FVC decline from 24 to 120 weeks were also comparable between the tiotropium Respimat® 5 μg, 2.5 μg, and HandiHaler® 18 μg groups: 35, 50, and 52 mL/year, as were those for the FEV_1_/FVC ratio (from 0.24 to 0.57 %) (Table 2).

**Table 2:** Rate of decline in FEV_1_, FVC, and FEV_1_/FVC ratio

	Tiotropium Respimat® 5 μg (n = 461)	Tiotropium Respimat® 2.5 μg (n = 464)	Tiotropium HandiHaler® 18 μg (n = 445)
Rate of decline in FEV_1_			
Annual rate of decline, mL	26	40	34
Difference to HandiHaler® 18 μg, mL (95 % CI)	−8 (−26,10); p = 0.3771	5 (−12, 23); p = 0.5537	
Difference to Respimat® 5 μg, mL (95 % CI)		13 (−4, 31); p = 0.1350	
Rate of decline in FVC			
Annual rate of decline, mL	35	50	52
Difference to HandiHaler® 18 μg, mL (95 % CI)	−17 (−48, 13); p = 0.2627	−3 (−33, 28); p = 0.8549	
Difference to Respimat® 5 μg, mL (95 % CI)		15 (−15, 44); p = 0.3423	
Rate of decline in FEV_1_/FVC			
Annual rate of decline, %	0.29	0.57	0.24
Difference to HandiHaler® 18 μg, % (95 % CI)	0.04 (−0.42, 0.50); p = 0.8543	0.33 (−0.13, 0.78); p = 0.1627	
Difference to Respimat® 5 μg, % (95 % CI)		0.28 (−0.17, 0.74); p = 0.2191	

Note that rounding of decimals has been applied throughout, including for the difference calculations

*Abbreviations*: *CI* confidence interval, *FEV*_*1*_ forced expiratory volume in 1 second, *FVC* forced vital capacity

**Fig. 3:**
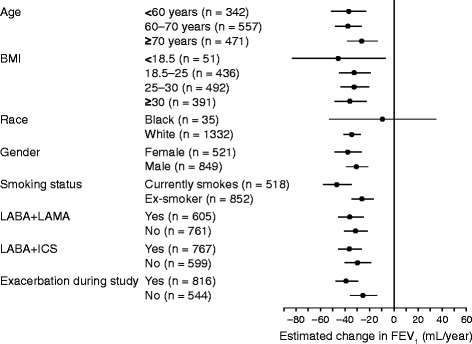
Forest plot of annual rate of forced expiratory volume in 1 second (FEV_1_) decline in lung function by baseline characteristics. *Abbreviations*: BMI = body mass index; FEV_1_ = forced expiratory volume in 1 second; ICS = inhaled corticosteroid; LABA = long-acting β_2_-agonist; LAMA = long-acting muscarinic receptor antagonist

Mean annual changes in FEV_1_, FVC, and FEV_1_/FVC by baseline characteristics for the substudy population (treatment arms pooled) are shown in Fig. 3 (FEV_1_) and in the Additional file 2: Figure S1 (FVC and FEV_1_/FVC). Similar declines in lung function were observed across most subgroups. The declines in FEV_1_ and FVC appeared slightly higher in patients who were current smokers versus ex-smokers; in patients receiving a long-acting beta-adrenergic bronchodilator combined with a long-acting muscarinic antagonist/inhaled corticosteroid at baseline compared with those not receiving these medications; and in the subgroup of patients experiencing an exacerbation during the study (vs. no exacerbation). A relatively high rate of decline was observed for patients with a low body mass index (BMI), and minimal decline in patients of Black race; however, these subgroups had small sample sizes (n = 51 and n = 35, respectively) and wide CIs. The proportion of Black patients was higher (2.6 %) in the spirometry substudy than in the overall TIOSPIR® population (1.5 %).

Based on lung function severity at baseline, the rate of decline of FEV_1_ for patients from GOLD Stages I + II (GOLD I, n = 4; GOLD II, n = 632) was larger than for patients from GOLD Stages III + IV (GOLD III, n = 580; GOLD IV, n = 130) (46 vs. 23 mL/year, respectively; difference [95 % CI]: 23 [9, 38]; p = 0.0017) (Table 3). The rates of decline of FVC and FEV_1_/FVC were similar for patients from GOLD Stages I + II and III + IV (Table 3).

**Table 3:** Rate of decline in FEV_1_, FVC, and FEV_1_/FVC ratio by GOLD stage

	GOLD I + II (n = 636)	GOLD III + IV (n = 710)
Rate of decline in FEV_1_		
Annual rate of decline, mL	46	23
Difference, mL (95 % CI)	23 (9, 38); p = 0.0017	
Rate of decline in FVC		
Annual rate of decline, mL	55	37
Difference, mL (95 % CI)	18 (−7, 43); p = 0.1658	
Rate of decline in FEV_1_/FVC		
Annual rate of decline, %	0.51	0.26
Difference, % (95 % CI)	0.25 (−0.12, 0.62); p = 0.1914	

Note that rounding of decimals has been applied throughout, including for the difference calculations

*Abbreviations*: *CI* confidence interval, *FEV*_*1*_ forced expiratory volume in 1 second, *FVC* forced vital capacity, *GOLD* Global Initiative for Chronic Obstructive Lung Disease

## Discussion

Results from the TIOSPIR® trial showed that tiotropium HandiHaler® and tiotropium Respimat® exhibit similar safety and efficacy profiles in patients with COPD [6, 7]. This included no differences in mortality or exacerbation efficacy. As previously reported, the prespecified analysis of trough FEV_1_ in the spirometry substudy showed that Respimat® 5 μg was noninferior to HandiHaler® (difference [95 % CI]: −10 mL [−38, 18]), but noninferiority was not shown for Respimat® 2.5 μg [7]. Patients in the substudy received similar baseline respiratory therapy.

The TIOSPIR® spirometry substudy provided an opportunity to examine further lung function outcomes with tiotropium HandiHaler® and Respimat®. Our results also showed no significant difference in the improvement from baseline in trough FVC with tiotropium Respimat® 5 μg or tiotropium HandiHaler®. Similar trends in both FEV_1_ and FVC increases over baseline were observed over time (from 24 to 120 weeks) in the tiotropium Respimat® 5 μg and HandiHaler® arms, suggesting that there is no tachyphylaxis following treatment, and supporting previous 1-year trial results [3, 10]. The improvements in FEV_1_ and FVC with tiotropium Respimat® 2.5 μg were, however, consistently lower than those for Respimat® 5 μg or HandiHaler® 18 μg, validating the higher tiotropium doses as those that are approved and available [11, 12].

The spirometry substudy of TIOSPIR® is the first to report rates of lung function decline for tiotropium Respimat®. Annual rates of FEV_1_, FVC, and FEV_1_/FVC decline were similar for tiotropium HandiHaler® 18 μg and Respimat® at the 5 μg or 2.5 μg daily dose. The annual rate of decline in FEV_1_ in the TIOSPIR® substudy was 26 mL for tiotropium Respimat® 5 μg and 34 mL for HandiHaler® 18 μg. These findings are comparable to the annual rate of decline in FEV_1_ that was reported in previous studies [4, 13, 14], even though this substudy included a smaller number of patients and evaluated them over a shorter time period. The annual rate of decline in post-bronchodilator FEV_1_ in large clinical trials of COPD appears to have reduced in recent years: UPLIFT® (2008) and Towards a Revolution in COPD Health (TORCH) (2007) exhibited rates of decline of 40 and 55 mL/year for the placebo groups, respectively (and 39–42 mL/year for the treatment groups) [4, 13], whereas the Evaluation of COPD Longitudinally to Identify Predictive Surrogate Endpoints (ECLIPSE) (2011) trial reported a rate of FEV_1_ decline of 33 mL/year [14]. The reduction in the rate of lung function decline may reflect improvements in COPD treatments and, at least in the case of the UPLIFT® trial, the use of other concomitant COPD medications in the placebo arm.

It has previously been reported that the rate of decline in FEV_1_ in patients with COPD is highly variable, and that rates of decline are increased among current smokers, patients with bronchodilator reversibility, and patients with emphysema [14]. Rates of decline in trough FEV_1_ and FVC among the TIOSPIR® spirometry substudy population appeared higher among current smokers, in those receiving a long-acting beta-adrenergic bronchodilator combined with a long-acting muscarinic antagonist/inhaled corticosteroid at baseline, and in those experiencing an exacerbation during the study. It remains to be determined whether the relative change in FEV_1_ could be used as a predictor for subsequent exacerbations or whether an improvement in FEV_1_ could be related to a reduction in exacerbation rate.

The rate of FEV_1_ decline reflects the composition of the population and was less marked in more severe patients. When the mean rate of FEV_1_ decline in the substudy population was calculated by disease severity (GOLD Stages I + II vs. III + IV), it was shown that the patients with less severe disease (GOLD I + II) exhibited a significantly faster annual rate of decline (46 mL) than those with more severe disease (GOLD III + IV; 23 mL; *p* = 0.0017). This is in contrast with the landmark study of Fletcher and Peto (1977) [15], which described that the rate of decline (“slope”) of FEV_1_ was increased with time in susceptible smokers (showing a steeper decline with lower FEV_1_), although this study did not include many patients with severe disease. However, Tantucci and Modina reviewed spirometric data of COPD patients included in the placebo arms of recent clinical trials to assess the lung function decline at different GOLD stages, and found that the loss of lung function, assessed as FEV_1_ decline expiratory airflow reduction, seems accelerated and therefore more relevant in the initial phases of COPD (in particular GOLD Stage II), where FEV_1_ is higher [16]. The results seen in the TIOSPIR® substudy population support this observation. In the UPLIFT® study of tiotropium HandiHaler® 18 μg versus placebo, which was performed in 5992 patients over 4 years, the rate of FEV_1_ decline also decreased with increasing disease severity (mean FEV_1_ decline of 49, 38 and 23 mL/year for GOLD Stages II, III, and IV, respectively) [16, 17]. Similar to a previous post-hoc analysis [18], the rate of FVC decline of patients in GOLD Stages I + II showed a trend toward being higher than that in GOLD III + IV patients in this substudy, but this did not reach statistical significance. We speculate that the loss of lung function in GOLD III and IV patients is likely to result from worsening air-trapping (increase in residual volume) as opposed to worsening airflow obstruction and expiratory flow limitation. Therefore, the rates of change of FVC and of the FEV_1_/FVC ratio are similar in the higher GOLD stages, while the loss of FEV_1_ is slower, and the overall change in the FEV_1_/FVC ratio reflects this composite effect.

A limitation of this study is that TIOSPIR® did not include a placebo arm. It was, however, designed as such because high adherence and follow-up would have been difficult to achieve in a placebo group without effective symptom relief, and since tiotropium HandiHaler® 18 μg was previously shown to be associated with reduced on-treatment mortality compared with placebo [4, 19, 20], it was used as a control. Another limitation is the fact that although the TIOSPIR® study itself was very large (N = 17,135), the spirometry substudy included only 1370 patients. Nonetheless, the rate of decline observed in this study is within the same range as that observed in previous larger studies [4, 13, 14], validating the result in which there was no difference between the three treatment arms. Additionally, the analyses of rates of decline were post-hoc, and the trial was not designed to calculate rates of decline. As described in the Methods, rates of decline were calculated using data from Week 24 as an anchor, and the decline was calculated every 24 weeks afterwards. Strengths of the study include the use of spirometry centers with high expertise in performing PFTs, across nine countries and 112 sites. Furthermore, the period analyzed, although not as long as in the UPLIFT and TORCH studies (4 and 3 years, respectively), was substantial at over 2 years.

## Conclusions

The TIOSPIR® spirometry substudy showed that tiotropium Respimat® 5 μg was noninferior to tiotropium HandiHaler® 18 μg for FEV_1_ (average 24–120 weeks), but Respimat® 2.5 μg was not. Tiotropium Respimat® 5 μg provided similar bronchodilator efficacy to tiotropium HandiHaler® 18 μg, with no significant differences in FVC and similar rates of decline of FEV_1_. When treatment arms were pooled, the rate of FEV_1_ decline among patients with less severe disease (GOLD Stages I + II) was larger than for patients with more severe disease (GOLD Stages III + IV), consistent with other recent studies.

## Additional files

Additional file 1: Table S1. Baseline characteristics by study population. (DOCX 16 kb) Additional file 2: Figure S1. Forest plot of annual rate of FVC (A) and FEV_1_/FVC (B) decline in lung function by baseline characteristics. (DOCX108 kb)

## Abbreviations

BMI: body mass index
CI: confidence interval
COPD: chronic obstructive pulmonary disease
ECLIPSE: Evaluation of COPD Longitudinally to Identify Predictive Surrogate Endpoints
FEV_1_: forced expiratory volume in 1 second
FVC: forced vital capacity
GLI: Global Lungs Initiative
GOLD: Global Initiative for Chronic Obstructive Lung Disease
HRQoL: health related quality of life
ICS: inhaled corticosteroid
LABA: long-acting β2-agonist
MMRM: mixed model repeated measures
PFT: pulmonary function test
SMI: Soft Mist™ Inhaler
TIOSPIR®: Tiotropium Safety and Performance in Respimat®
TORCH: Towards a Revolution in COPD Health
UPLIFT®: Understanding Potential Long-term Impacts on Function with Tiotropium
