# Supplementary material for: The Tiotropium Safety and Performance in Respimat® (TIOSPIR®) Trial: Spirometry Outcomes
Source: Respir Res. 2015 Sep 15;16(1):107. doi: 10.1186/s12931-015-0269-4 (PMC4570597; doi:10.1186/s12931-015-0269-4)
Supplement: Additional file 1: Table S1. — Baseline characteristics by study population. (DOCX 16 kb) [file 12931_2015_269_MOESM1_ESM.docx]

**Supplementary Table S1. Baseline characteristics by study population**

|  | **Spirometry substudy**  **(n = 1370)** | **Non-substudy**  **(n = 15,746)** | **Total**  **(n = 17,116)** |
| --- | --- | --- | --- |
| Male sex, % | 62.0 | 72.3 | 71.5 |
| Race, %  White  Black  Asian  Missing* | 97.2  2.6  0.2  0.0 | 80.2  1.4  15.4  3.0 | 81.6  1.5  14.2  2.7 |
| Age, years | 65.6 ± 8.9 | 64.9 ± 9.1 | 65.0 ± 9.1 |
| Current smoker, % | 37.8 | 38.1 | 38.1 |
| Smoking history, pack-years | 50.3 ± 28.4 | 43.2 ± 24.3 | 43.8 ± 24.8 |
| Any respiratory medication, % | 96.1 | 90.1 | 90.6 |
| Anticholinergics^†^ | 69.3 | 58.2 | 59.1 |
| LABA^†^ | 66.9 | 61.4 | 61.8 |
| ICS^†^ | 61.1 | 58.8 | 59.0 |

*This category includes 468 patients from France where race cannot be collected as well as one patient from Peru whose race was determined to be Mestizo. ^†^Used alone or in combination.

*Abbreviations*: ICS = inhaled corticosteroid; LABA = long-acting β2-agonist.3
