# Supplementary material for: The Tiotropium Safety and Performance in Respimat® (TIOSPIR®) Trial: Spirometry Outcomes
Source: Respir Res. 2015 Sep 15;16(1):107. doi: 10.1186/s12931-015-0269-4 (PMC4570597; doi:10.1186/s12931-015-0269-4)
Supplement: Additional file 2: Figure S1. — Forest plot of annual rate of FVC (A) and FEV1/FVC (B) decline in lung function by baseline characteristics. (DOCX108 kb) [file 12931_2015_269_MOESM2_ESM.docx]

**

**

**Supplementary Figure S1. Forest plot of annual rate of FVC (A) and FEV_1_/FVC (B) decline in lung function by baseline characteristics**

*Abbreviations*: BMI = body mass index; FEV_1_ = forced expiratory volume in 1 second; FVC = forced vital capacity; ICS = inhaled corticosteroid; LABA = long-acting β_2_-agonist; LAMA = long-acting muscarinic receptor antagonist.
